# Lymphatic Vascular Response to Acute Inflammation

**DOI:** 10.1371/journal.pone.0076078

**Published:** 2013-09-27

**Authors:** Pier-Anne Lachance, Amy Hazen, Eva M. Sevick-Muraca

**Affiliations:** 1 The Center for Molecular Imaging, The Brown Foundation Institute of Molecular Medicine, The University of Texas Health Science Center, Houston, Texas, United States of America; 2 Department of Molecular Physiology and Biophysics, Baylor College of Medicine, Houston, Texas, United States of America; 3 IMM Flow Cytometry Center, The Brown Foundation Institute of Molecular Medicine, The University of Texas Health Science Center, Houston, Texas, United States of America; McGill University, Canada

## Abstract

During acute inflammation, functioning lymphatics are believed to reduce edema and to provide a transiting route for immune cells, but the extent at which the dermal lymphatic remodeling impacts lymphatic transport or the factors regulating these changes remains unclear. Herein we quantify the increase in lymphatic endothelial cells (LECs) and examine the expression of pro-angiogenenic and lymphangiogenic factors during acute cutaneous hypersensitivity (CHS). We found that LECs actively proliferate during CHS but that this proliferation does not affect the lymphatic vessel density. Instead, lymphatic remodeling is accompanied by lymphatic vessel leakiness and lower ejection of lymph fluid, which is observed only in the proximal lymphatic vessel draining the inflamed area. LECs and the immune cells release growth factors and cytokines during inflammation, which impact the lymphatic microenvironment and function. We identified that FGF-2, PLGF-2, HGF, EGF, and KC/CXCL17 are differentially expressed within tissues during acute CHS, but both VEGF-C and VEGF-D levels do not significantly change. Our results indicate that VEGF-C and VEGF-D are not the only players and other factors may be responsible for the LECs proliferation and altered lymphatic function in acute CHS.

## Introduction

The processes of angiogenesis and lymphangiogenesis are recognized to play critical roles in cancer progression, metastasis, and many chronic diseases, but also serve non-pathological roles in the normal progression and resolution of inflammation [1]. During inflammation, functioning lymphatics are believed to reduce edema and to provide a transiting route for immune cells; but whether extensive dermal lymphatic remodeling that presumably takes place in inflamed tissue directly impacts transport remains unclear. Indeed, using near-infrared fluorescence (NIRF) lymphatic imaging, we and others have noted both dense hyperplasia and hypoplasia of dermal lymphatics in the arms and legs of lymphedema subjects with profound edema, suggesting a more complex relationship between dermal lymphatic remodeling and effective lymphatic transport [2,3]. Whether the dermal lymphatic remodeling directly benefits regional lymphatic transport and edema reduction, or whether other factors are responsible, remains to be investigated.

Because the vascular endothelial growth factor (VEGF) family of ligands and receptors are known to promote both hemovascular and lymphatic proliferation, several investigations have focused on their role in vascular remodeling during acute and chronic skin inflammation [4–7]. VEGF-C signaling through VEGFR3 is required for embryonic lymphangiogenesis and therefore believed to be a major player in adult lymphatic remodeling [1]. VEGF-C expression in the skin reduces inflammatory symptoms of cutaneous hypersensitivity (CHS) in mice and has been postulated to decrease edema and the inflammatory response to CHS by specifically enhancing the lymphatic proliferation [4]. Other members of the VEGF family (such as VEGF-A and VEGF-D) induce lymphangiogenesis in vivo through their interaction with VEGFR3, but each has different effects on CHS [8,9]. In contrast to evidence that lymphangiogenesis mediates edema, Huggenberger et al. used Evans blue dye to show that no changes in ear lymphatic drainage patterns occur in acute CHS reactions despite the observation that edema accompanying CHS is reduced in K14-VEGF-C and K14-VEGF-D overexpressing mice compared to control mice [5]. Thus, while VEGFR-3 mediated lymphangiogenesis has been studied as part of the initiation and resolution of inflammation, the role of effectors of lymphatic function has yet to be investigated with non-invasive techniques that (i) directly and longitudinally assess changes in lymphatic function and (ii) enable correlation to the expression of immune and other stimulatory factors that impact the contraction and ejection fraction of the “pumping” lymphatic system.

To investigate the role of lymphatic function in acute inflammation, we induced acute CHS reactions in normal mice and longitudinally imaged the dermal lymphatics afferent, and the contractile, conducting lymphatics efferent to the regional draining lymph node using NIRF lymphatic imaging. We evaluated the population of cells in inflamed skin, using flow cytometry and histology, as a function of time after induction of acute CHS reaction and assessed lymphatic endothelial cell (LEC) proliferation using a method of incorporation to directly assess proliferation quantitatively. We also assessed the tissue protein expression of growth factors associated to angiogenesis, lymphangiogenesis, and inflammation.

Our results show that the LECs proliferate in early onset of CHS, but that this proliferation is not sustained from day 4 to 7 post-CHS. We also determined that the efferent lymph transport is impaired in early CHS as shown by a reduction in lymph ejection, but not by a reduction in lymphatic vessel contractility in both the inflamed and contralateral untreated sides of animals. We then identified several growth factors and inflammatory molecules to be differentially expressed in early inflammation as compared to non-treated animals and animals at later stages of the CHS reaction. VEGF-C and VEGF-D expression were not significantly changed during the course of the CHS reaction. These data may indicate that multiple growth factors other than VEGF-C and VEGF-D work in concordance with VEGF family to promote LECs proliferation, regression, and mediate lymph flow for resolution of CHS inflammation.

## Materials and Methods

### Ethics Statement

This study was performed in strict accordance with animal use protocols approved by The Animal Welfare Committee at the University of Texas Health Science Center (IACUC, protocol number AWC 12-015). Mice were euthanized if they met any early removal criteria (lethargy, hunched posture, or ruffled coat) to limit suffering.

### Induction of cutaneous delayed-type hypersensitivity (CHS) reaction and EdU injections

CHS reactions were induced in wild type C57B/6 mice. For all studies, mice were sensitized by topical application on the shaved upper back of 50µl 2% oxazolone (4-Ethoxymethylene-2-phenyl-2-oxazolin-5-one) (Sigma, St Louis, MO) in acetone: olive oil (4:1 vol/vol). Five days after sensitization (day 0), mice were challenged with 100µl 1% oxazolone in solution on the shaved lower back. To assess cell proliferation, 200µg of EdU (5-ethynyl-2’-deoxyuridine) (Invitrogen Corporation, Carlsband, CA) in 200µl PBS was injected IP in challenged mice and in unchallenged mice every 24 hour for 3 days from day 0. All animal studies were approved by the Animal Welfare Committee at the University of Texas Health Science Center.

### Isolation of cells from mouse tissue, fluorescence-activated cell sorting, and flow cytometry analysis

Mice were humanly euthanized at day 0, 3, or 7 days post-CHS challenge. Skin tissue was removed and placed in 0.1% trypsin in HSBS with 1% FBS for 30 minutes at room temperature. The dermis was then separated from the epidermis using forceps and scissors and minced in 1mm sections before being incubated in 1mg/ml collagenase/dispase (Roche, Indianapolis, IN) in HSBS with 5% FBS at 37°C for 45 minutes on a shaking platform. After incubation, the dermis samples were placed on ice. The dermis was passed through a 70µm tissue sieve using a rubber plunger. The sieve was rinsed multiple times with PBS. The collected cell mixture was then centrifuged to form a cell pellet. Cells were washed once with staining buffer (5% FBS in HSBS with 5mM EDTA) and then resuspended in staining buffer with of FC block (BD Bioscience, Franklin Lakes, NJ) before incubated with fluorescence conjugated primary antibodies against CD45 (clone 30-F11) (Ebioscience, San Diego, CA), CD31 (clone 390) (Ebioscience), podoplanin (clone eBio8.1.1) (Ebioscience), and Calcein AM 10µM (BD Bioscience) for 30 minutes on ice. We selected the markers CD31 and podoplanin to identify LECs [10]. Adequate sampling of LECs was indicated with LEC counts >300.

For cell proliferation analysis, cell isolation was performed as described above, but samples were enriched for CD31 positive cells before staining. The sample was incubated for 30 minutes on ice with biotin conjugated CD31 clone 390 (Ebioscience). Streptavidin magnetic beads, MagCellect Streptavidin Ferrofluid (R&D Systems, Mineapolis, MN), were added to the cell mixture and incubated for 10 minutes on ice. The sample tube was placed in a magnet holder to allow separation. The supernatant was removed. CD31+ cells were washed and stained for CD45, podoplanin, and Calcein AM, and Streptavidin PE-CY7 (Ebioscience). Cells were sorted and collected in media with 20% FBS. After sorting, cells were fixed with PFA 2% for 5 minutes, permeabilized with saponin for 1minute, and stained for EdU intercalation using the Click-iT EdU Alexa Fluor 647 Flow Cytometry Assay kit (Invitrogen Corporation). Cells were resuspended in staining buffer with DAPI at 5µg/ml. In this case, DAPI was use to differential cells from debris that was accumulated during cell sorting. During the analysis, we used the total population of CD31+ cells as a control to set the positive gate for EdU, since we could obtain a larger sample size using this population. We slightly adjusted the gates in each individual experiment to allow for changes in staining efficiencies and digestions, but the gates within each experiments remained the same. All flow cytometry and fluorescence-activated cell sorting (FACS) was performed using the BD FACSAria™ II (BD Bioscience) and the BD FACSDiva software (BD Bioscience) and data analysis was performed using FlowJo version 7.5.6 (Tree Star, Ashland, OR) (see Figure S1).

### Immunoblot

Cells isolated by FACS were plated on a 24 well tissue culture plates coated with fibronectin (Sigma Aldrich) (5µg/ml) in ECM media (Sciencell Research Laboratories, Carlsbad, CA) enriched with 5% FBS, antibiotic, 100 units/mL of Penicillin, 100 µg/mL of Streptomycin, and Endothelial Cell Growth Supplement (Sciencell Research Laboratories) in a humidified incubator at 37°C and 5% CO_2_. The media was changed after the cells adhered to the plate (5 hours). Cells were cultured for 2 days before being lysed using RIPPA buffer (1% NP-40, 0.5% sodium deoxycholate, 0.1% SDS,0.15M NaCl, 2mM EDTA in Tris base). Protein lysates were quantified using BCA assay (Thermo, Fisher Science, Rockford, IL). Membrane was blotted with antibodies against PROX-1 (Relia Tech, Germany), LYVE-1 (Abcam, Cambridge, MA), and GAPDH (Cell Signaling, Danvers, MA). Secondary detection was performed using antibodies conjugated with IRDye800 (Li-Cor Biosciences, Lincoln, NB) and detected by the Odyssey system (Li-Cor Biosciences) imager for near-infrared fluorescence.

### Immunohistochemistry

Cells isolated by FACS were placed on tissue culture plates coated with fibronectin (5µg/ml) (Sigma Aldrich). Cells were grown in ECM media for 2 days before being trypsinized and plated on culture slides (BD Bioscience). Cells were fixed with 4% PFA and permeabilized with 0.1% tritonX. After PBS washes, blocking buffer (5% donkey serum in PBS with 1% BSA) was added to the well and incubated for 1 hour at RT. Primary antibodies to PROX-1(Relia Tech) and Podoplanin (Santa Cruz Biotechnology, Santa Cruz, California) were diluted in blocking buffer and incubated in the wells overnight at 4°C. Cells were then stained with secondary antibody conjugated to fluorescent dye for detection. Microscopy was then performed using Leica DM6000B/TCS SP5.

Paraffin-embedded sections from mouse whole skin were sectioned at 7µM and de-paraffinized using Xylene 100% for 3 minutes twice and then rehydrated using a gradient of ethanol. Heat antigen retrival was performed at 100°C for 30 minutes in calcium citrate buffer 10mM at pH 6. Sections were first incubated in blocking buffer for 1hour and then stained overnight at 4°C with podoplanin primary antibody (Santa Cruz Biotechnology). After washing, sections were incubated with secondary anti-hamster alexa-546 (Invitrogen) for 2 hours at room temperature. Stained sections were imaged (Leica DM600B) and analyzed using the Surveyor Software (Objective Imaging Ltd, Cambridge, UK). To measure lymphatic density, 5 random regions of interest of 100µm x 100µm were selected. Each region encompassed the epidermis. We counted the number of vessels positive for Podoplanin staining counted. A vessel was defined as a structure with a lumen and at least one DAPI positive nucleus within the structure. We also used ImageJ to determine the cross sectional area of the each vessel.

### Tube formation Assay

Wells of a 96-well tissue coated plate were coated with 20µl of Matrigel (BD Bioscience). Matrigel was allowed to become solid at 37°C for 30 minutes. Cells isolated by FACS were diluted in ECM media and 5 x 10^4^ cells were plated in each well. Plates were incubated at 37°C in a humidified incubator for 24 hours. Bright field microscopy was performed at that time to assess structures using a Leica DM IL microscope.

### Lymphatic imaging

NIRF lymphatic imaging was conducted to determine whether there was a change in architecture of the lymphatic vessels and to assess changes in efferent lymphatic function from the draining lymph nodes on the side of inflammation and on the untreated, contralateral side of the same mouse, in order to determine whether the effects were systemic. Wild type C57B/6 mice were imaged before the sensitization, at day3 and day 7 post topical application on the right thigh of the mouse. This area was selected because it drains its lymph into the inguinal lymph node, as does the lymph from the tail, and therefore allowed us to examine the effect of inflammation on the lymphatic vasculature afferent to the draining lymph nodes without having to inject dye directly into the inflamed skin. For a detailed protocol of mouse lymphatic imaging see Robinson et al [11]. Briefly, mice were anesthetized using isoflurane and with a 31-gauge needle, 5 µL to 10 µL of ICGreen (Akorn Inc., Lake Forest, IL) were injected ID at 5mg/ml in the left and right side of the base of the tail. The animals were then imaged using a custom NIRF imaging system. Image integration times of 200ms were used and 1000 image frames were analyzed to quantify propulsive lymphatic flow function on each lateral side of the mice. ImageJ and Matlab were used to analyze the images. To determine the effect of CHS on the lymphatic flow of the conducting vessel connecting the inguinal and the axillary lymph node (LN)s was measured through counting the number of dye “packets” transiting collecting vessels for the duration of imaging [12]. The plot of fluorescence intensity versus time and length along the lymphatic vessel enabled spatio-temporal assessment of distal and proximal lymphatic propulsive flow. We used these plots to determine the lymphatic velocity, the time a packet moved from the inguinal lymph node to the axillary LN after a contraction, and the time in which the vessels are saturated with dye. These measurements were taken for a series of N>10 pulses per animal per group.

### Measurement of angiogenic and lymphangiogenenic factor

Inflamed skin was harvested from untreated mice as well as mice 3 and 7 days post-CHS. The dermis was separated from the epidermis and the dermis was homogenized in RIPA buffer with protease inhibitors and phosphatase inhibitors. Protein levels were quantified using the MILLIPLEX Mouse Angiogenesis/Growth Factor Panel (MAGPMAG-24K) (EMD Millipore) and protocol provided by the manufacturer. Analysis was performed on a Luminex 200 (Luminex Corp., Austin, TX) and used the Xpotent 3.1.871.0 Software (Luminex Corp., Austin, TX) and the concentration of each analyte was determined using a log regression curve and a standard curve. The assay was performed in quadruplicate on each sample and the concentration of analytes and p values of one representative replicate were reported.

### Statistical Analyses

Statistical analyses were performed using R and excel statistical functions (Microsoft) [13]. Groups were compared using the non-parametric Kruskall Wallis test or student T test or ANOVA where appropriate. Experiments involving multiple groups were first compared using ANOVA followed by T test for comparing differences between specific relevant groups. P-values were corrected within each experiment using false discovery rate (FDR) correction. Comparisons were considered significant if p-value < 0.05 and FDR < 0.1.

## Results

### Identification of lymphatic endothelial cell in normal mouse

**Figure 1:**
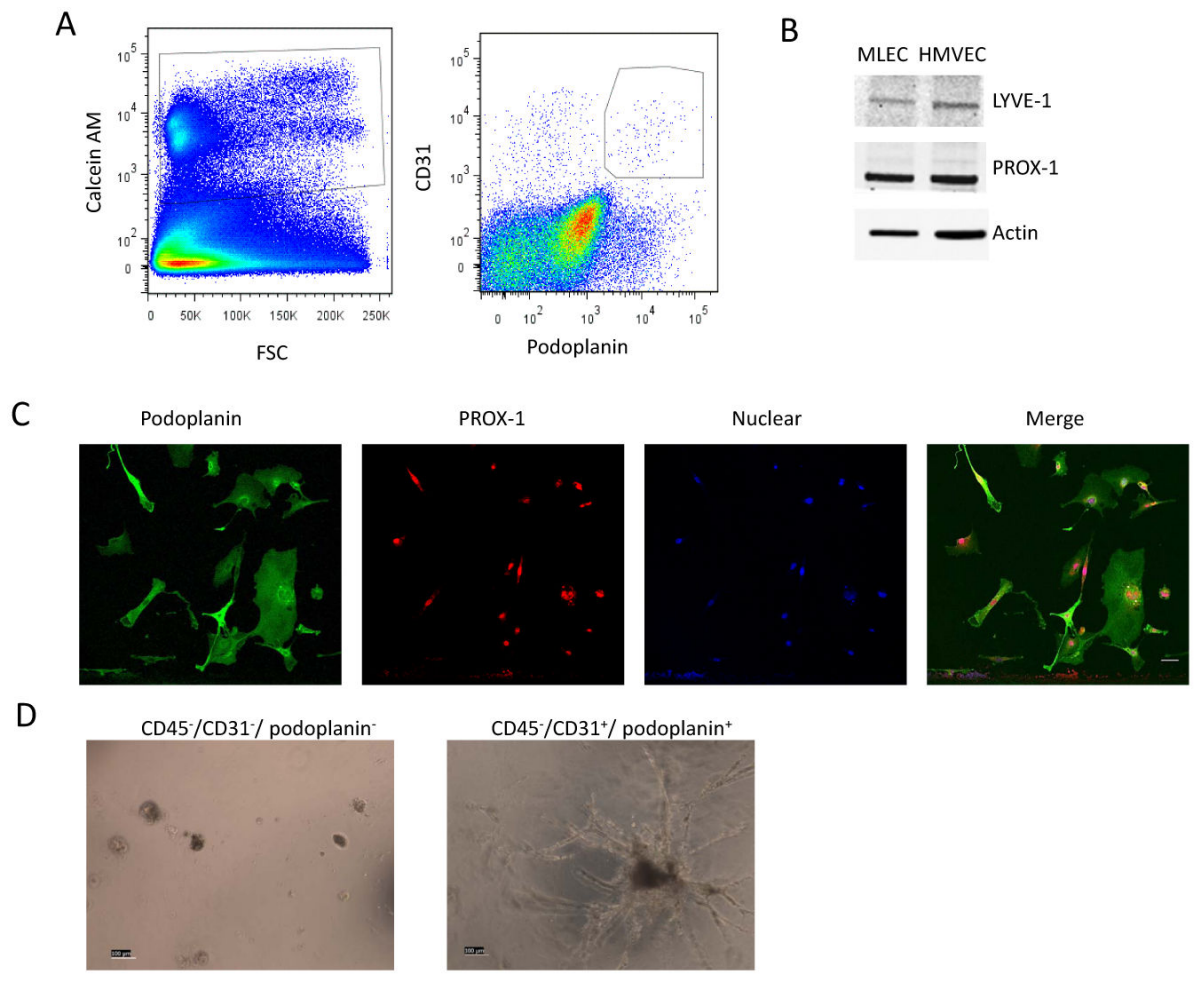
Isolation of lymphatic endothelial cell population. FACS was performed on cells isolated from the back skin of wild type mice. (**A**) Events are first gated on FSC and SSC for debris. The gated events were then plotted to determine Calcein Am incorporation, which will occur only in live cells. We observed a variation in levels of Calcein incorporation in different cell type as shown by the multiple populations observed. We then gated the positive events for caicein Am as shown in the left plot. The right plot shows the viable cells plotted for expression of CD31 and podoplanin, which we identified to be LEC population. (**B**) Western blotting for PROX-1 and LYVE-1 on cell lysate from isolated CD45^-^/CD31^+^/ podoplanin^+^ population and cultured human microvascular lymphatic endothelial cell (HMVEC) shows comparable expression of these lymphatic endothelial markers in both cell types. (**C**) Isolated CD45^-^/CD31^+^/ podoplanin^+^ cells were cultured and stained for podoplanin and PROX-1. Immunofluorecscent image of the cells is shown. Scale bar 100µm. (**D**) Isolated CD45^-^/CD31^+^/ podoplanin^+^ cells and CD45^-^/CD31^-^/ podoplanin^-^ were seeded on Matrigel for 24hours and only CD45^-^/CD31^+^/ podoplanin^+^ cells were able to form tube like structures. Scale bar 100µm.

LECs markers have been identified in the last 15 years. These markers have been used to isolate LECs from human and murine dermal tissue in the past [5,14,15]. We aimed to identify the relative changes in cell populations within mouse skin. CD45 was used as a marker for the identification of leukocytes and CD31+/podplanin+ were used as markers for the LEC population. Unlike the work of others, we employed the viability dye Calcein AM to differentiate the non-cellular events expected from collagenous tissues from the events that arose from viable cells before gating on FCS-A/SSC-A dot plot. It is noteworthy that, more than 50% of the events in the CD45-/CD31+/podoplanin+ gate were found to be non-viable events (Figure S1), indicating the need to use viability dyes to obtain accurate LEC percentages from skin samples. After selecting for live cells, we isolated by FACS the events that were negative for CD45 and positive for both CD31 and podoplanin (Figure 1A). These FACS isolated cells accounted for 1.16% of the CD45- cells in untreated, wild type C57BL\6 mice skin.

Immunoblot for PROX-1 and LYVE-1 showed comparable expression levels in the FACS isolated cells and cultured human dermal microvascular lymphatic endothelial cells (Figure 1B). Immunostaining of the FACS isolated cells also showed nuclear expression of PROX-1 and membrane staining for podoplanin consistent with LEC identity (Figure 1C). Finally, the FACS isolated cells formed tube structures on matrigel within 24 hours while the control cells which were CD45-/CD31-/Podoplanin- did not (Figure 1D). Together, this data indicated that the isolated cell population was of LECs origin.

### Identification of changes in cell populations of the skin in CHS reactions

**Figure 2:**
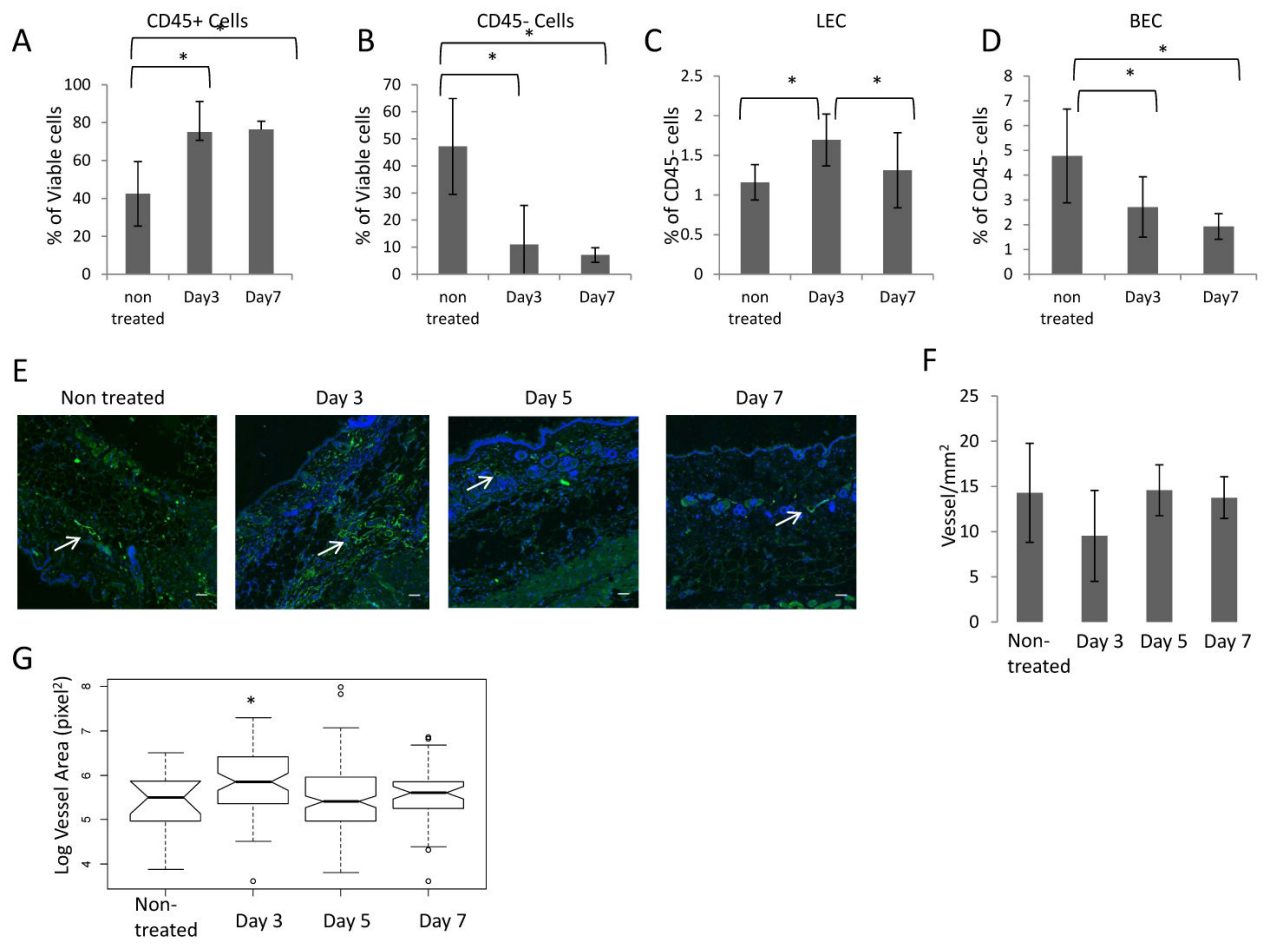
Quantification of LECs *in vivo*. (**A**-**C**) Single cell suspension was isolated from non-treated wild type mice, 3, or 7 days post CHS (N>5). Viable/CD45- , viable/CD45^+^, viable/CD45^-^/ CD31^+^/podoplanin^+^, and viable/CD45^-^/ CD31^+^ cells were quantified using flow cytometry analysis and the FLOWJo software. We observed that the percentage of viable CD45+ cells within the viable cell population in the skin of mice was elevated at days 3, and 7 post-CHS compared to non-treated animals (p<0.05). The percentage of the CD45- cell population within the viable population decreased at days 3 and day7 post-CHS compared to non-treated. Animals (p<0.05) We observed that the percentage of viable CD45^-^/CD31^+^/ podoplanin^+^ cells within the CD45- population increased significantly (p<0.05) at day 3 post CHS as compared to non-treated, and 7 days post CHS. (**E**) Immunofluorescence analysis was performed for podoplanin (green) and DAPI (blue) to identify lymphatic vessels in the skin. Arrows indicate location of lymphatic vessel. Scale bar 200µm. (**F**) Quantification of lymphatic vessels was performed using imageJ. Vessels were counted if a lumen was observed and if at least one nucleus was within the podoplanin signal. No significant change in the lymphatic density was observed. **G**) Quantification of the lymphatic vessel cross sectional area in pixel^2^. We found a significant difference in the size of the vessels at day 3 post-CHS as compared to the other time points. Significance (p<0.05) is denoted with a * and a bracket is used to identified the significantly different groups.

One of the hallmarks of CHS is the infiltration of leukocytes in the inflamed area at day 3 post-treatment. We observed a significant increase in leukocyte (CD45+) cells from 42% to 75% of the total viable cell population indicating that leukocytes infiltrated the inflamed skin (Figure 2A). Because of this large infiltration, there is a corresponding change in the proportion of non-leukocyte (CD45-) cell from 47% in non-treated skin to 12% of the total viable cell population at day 3 post CHS (Figure 2B). There were no significant changes in the percentage of both CD45+ and CD45- cells between day 3 post-CHS and day 7 post-CHS, indicating a limited change in the cellular composition of the skin between those time points.

We compared the relative changes in LECs percentage within the non-leukocyte cell population during CHS to determine if changes in LEC numbers could be observed using this method. The relative percentage of the non-leukocyte cell population provided a comparison to determine the relative abundance of LECs during treatment. Flow cytometry analysis of inflamed skin samples showed that the relative percentage of LECs (Calcein Am+/CD45-/CD31+/podoplanin+) within the non-leukocyte cells population increased from 1.16% to 1.69% at day 3 post-CHS as compared to non-treated mice, and reduced in percentages at day 7 post-CHS (1.3%), p<0.05 (Figure 2C). These results indicate that LEC population increases in relative abundance, within the non-leukocyte population, following the onset of acute inflammation. In contrast, we found that the relative percentage of non-leukocyte cells that were CD45-/CD31+/podoplanin- cells (or blood endothelial cells (BECs)) decreased significantly from non-treated skin (4.8%) to day3 (2.7%) and day 7 post-CHS (1.9%) without being significantly different between these latter two time points (Figure 2 D). It was previously observed by others that the blood vessel density is unchanged at day 2 of CHS, but that the blood vessels are dilated and leaky during CHS [16–19]. We therefore hypothesized that since the relative percentages of LECs increased, that the LEC population might be proliferating.

We found that the fluorescence intensity from CD31 and podoplanin antibodies that defined the LEC population remained constant with and without CHS treatment indicating that CD31 expression does not change on LECs with inflammation, in agreement with conclusions reached using other methods [20].

We measured the lymphatic density in the dermis by histology in non-treated animals, and at day 3, 5, and 7 post-CHS (Figure 2E). There was no significant difference in the lymphatic density between the time points (Figure 2E-F), consistent with the findings of others [4,6]. While many cells were stained for podoplanin at day 3 post-CHS, they were not within structures with a defined lumen. These cells might originate not only from LECs since we also observed by flow cytometry that populations of leukocyte cells also express podoplanin. We found that the cross sectional area of the vessels is significantly larger at day 3 post-CHS compared to the other days (Figure 2F).

### Measuring lymphatic endothelial cells proliferation in CHS reactions

**Figure 3:**
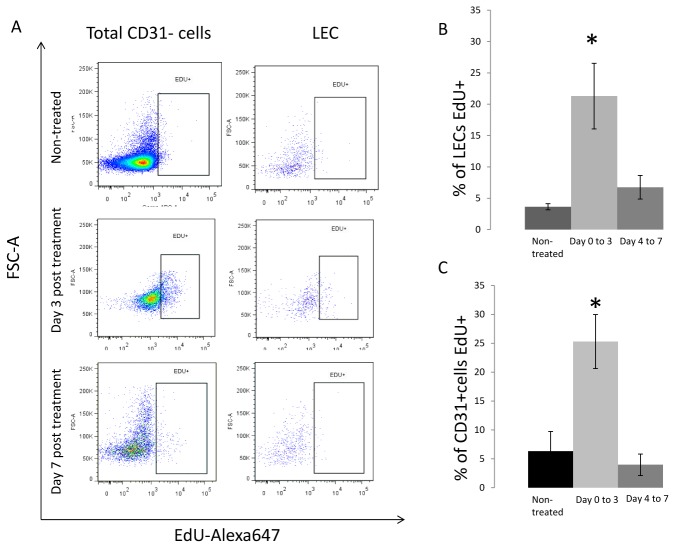
Identification of proliferating LECs. (**A**) EdU was injected every 24 hours I.P into mice either without treatment or after CHS. Single cell suspensions were made and viable CD45^-^/CD31^+^/ podoplanin^+^ cells were isolated using FACS. Cells were then fixed with 2% PFA, and stained using click it chemistry with an Alexa647 dye and DAPI. Flow cytometry analysis was performed on the sample to identify DAPI ^+^ /EdU ^+^ cell population. Sample flow cytometry plot of the FCS-A to EdU fluorescence for all three groups, non-treated, injected from day 0 to day3, and from day 4 to day 7. (**B**) Quantification of LECs or total CD31+ cells that stained for DAPI ^+^ /EdU ^+^ in non-treated mice, in mice injected from day 0 to day 3 post CHS, and in mice injected at day 4 to day 7. We observed a significant increase in the number of LECs and total CD31+ cells that intercalated EdU from day 0 to 3, the treated anmials with injection from day 4 to 7, and the non-treated groups (N=3) (p<0.001).

To determine whether the LECs undergo proliferation in the skin at day 3 of CHS as indicated by the histological observation and the relative abundance of LECs, we used EdU intercalation as a marker of cell proliferation [21,22]. We found that more than 20.45% of the LECs had incorporated EdU between onset of CHS and day 3 as compared to 4.25% in untreated mice (p<0.001) (Figure 3A-B). We also wanted to determine if LEC proliferation was restricted to the initial onset days of CHS and found that only 6.75% of the LEC were EdU positive if mice were injected with EdU from day 4 to day 7 post-CHS. We also measured the percentage of the total CD31+ cells (leukocyte and non-leukocytes) that intercalated EdU in the skin in order to determine the level of EdU fluorescence within proliferating cells. We observed a similar induction of cell proliferation in the total CD31+ population (Figure 3A,C). These data indicate that the observed increase in the relative percentage of LECs population may be responsible for changes in histological observation, and that vessel integrity might be due in part by a transient induction in LECs proliferation.

### Lymphatic flow measurement using near infrared dye ICG

**Figure 4:**
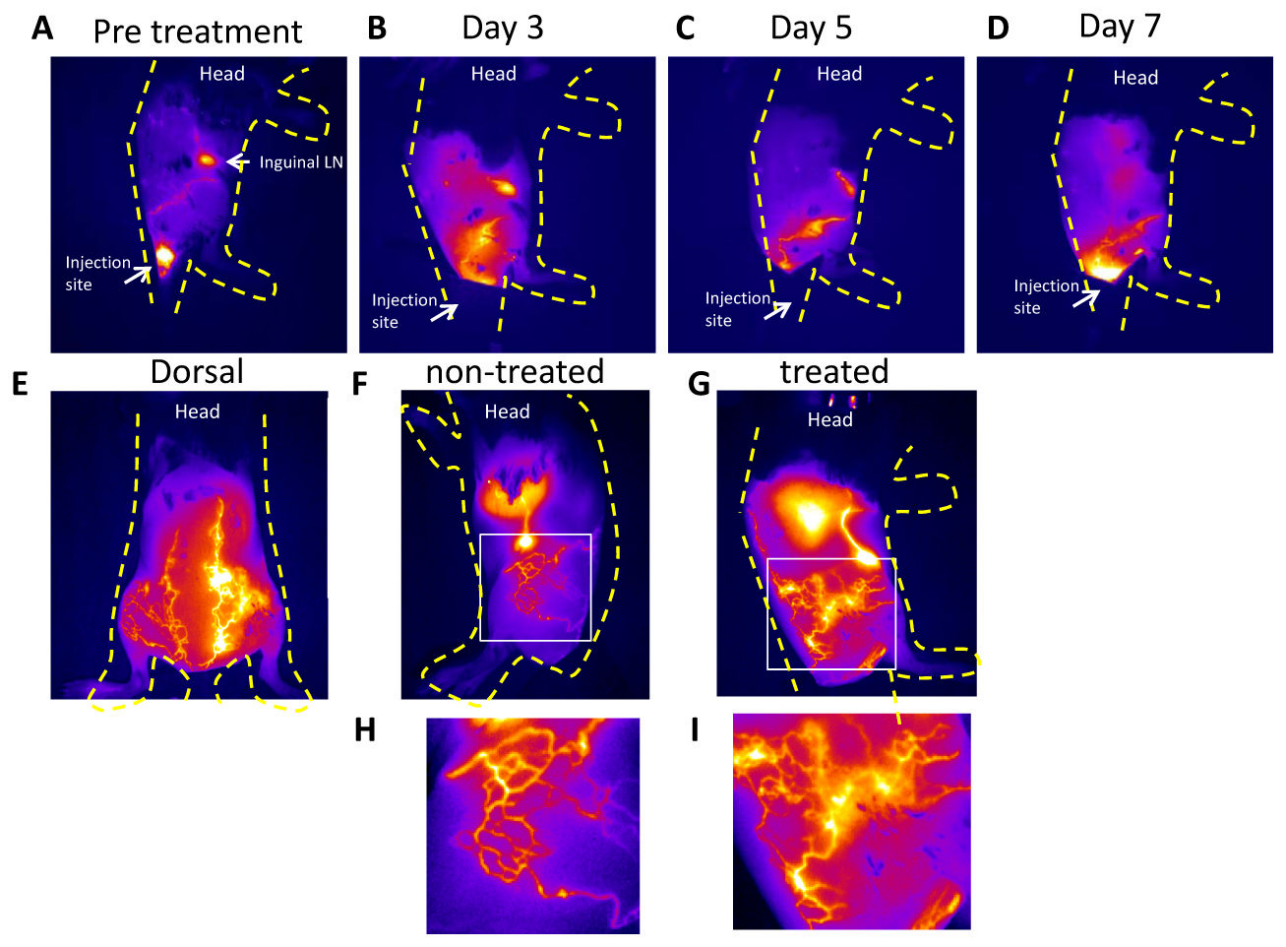
Lymphatic imaging using ICG. (**A**-**D**) Mice were injected I.D at the base of the tail with IGC to highlight lymphatic architecture at days 0, 3, 5, and 7 post-CHS treatment. We show images from one representative mouse. Oxazolone was applied on the right thigh of the mouse and the left side was left untreated. The yellow dotted line is an outline of the mouse body. We observed extravasation of the ICG dye after the onset of CHS. (**E**-**I**) We show images of a representative mouse at day 3 post-CHS to compare the changes occurring in the CHS treated side and non-treated side. The thigh area was increase in size to help visualize the leakiness of the dye in the interstitial area.

Lymphatic vessels are used for the infiltration of immune cells to the site of inflammation and enable their transport to regional LNs where the immune response occurs. We show in this study, as others have also shown, that lymphatic flow is perturbed during inflammation [4,23,24]. NIRF imaging results did not show changes in lymphatic architecture throughout CHS reaction. In the images taken pre-CHS, dye was well circumscribed within the vessels, but not at day 3, 5 and 7 post-CHS where imaging indicated dye extravasation (Figure 4 A-D). As prominently shown in the dorsal and lateral views of CHS treated and the non-treated sides of a mouse 3 days post-CHS, the lymphatic vessels “leaked” dye extravascularly, but still conducted lymph as shown by the ability of the dye to reach the axillary lymph node (Figure 4 E-I).

**Figure 5:**
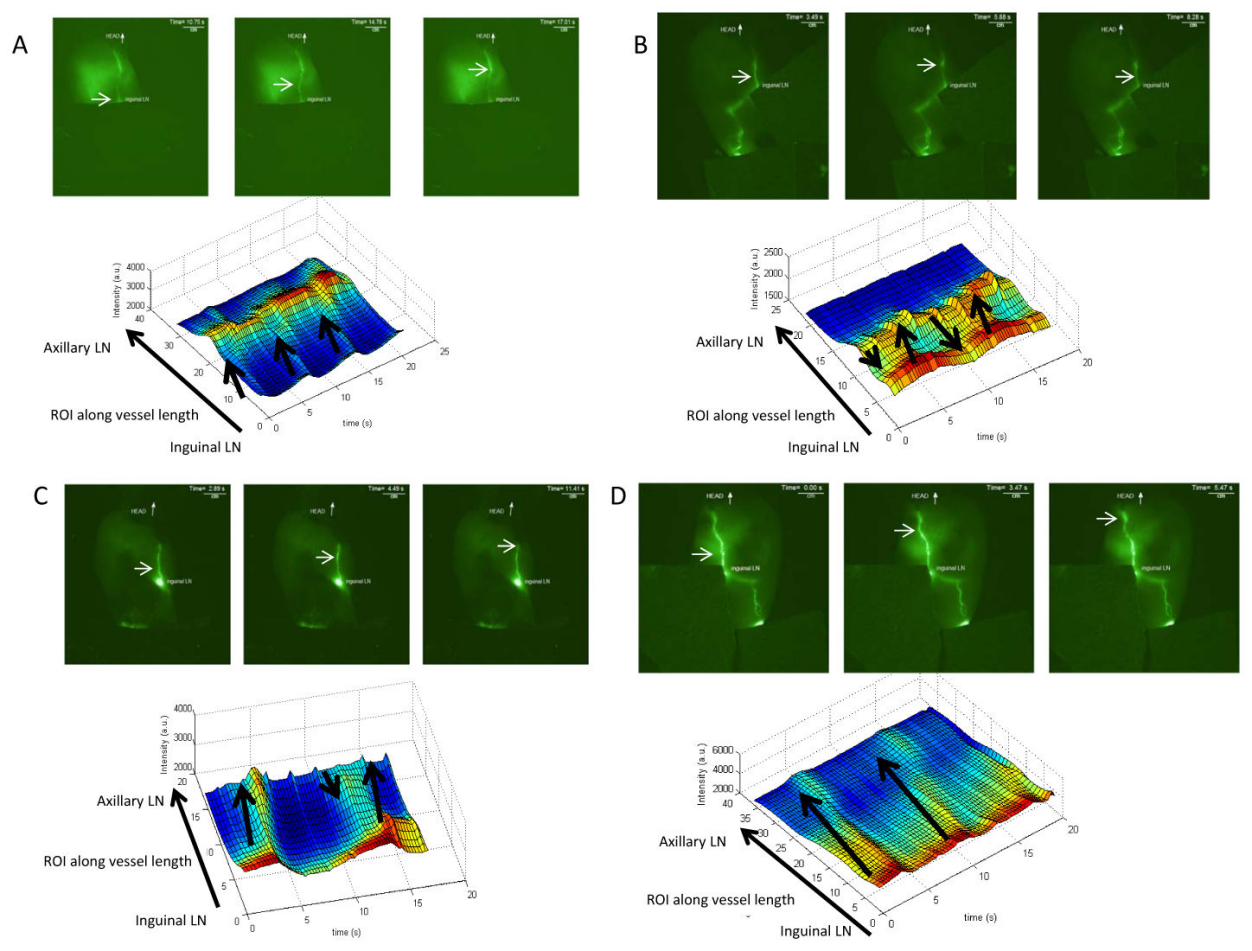
Lymphatic flow measurement using near infrared dye ICG. **A-D** Mice were injected I.D. with IGC at the base of the tail to quantify the contractility and qualify the ejection fraction of the lymph at days 0(**A**), 3(**B**), and 7(**C**) of post-CHS in the contralateral view at day 3(**D**) (N=3). Selected frames are showed from the same sample mice to illustrate the changes that occur in the lymphatic flow. Spatio-temporal fluorescent intensity maps are shown to visualize the ejection fraction of the ICG dye. We observed that mice pre-treatment (**A**), at day 7 post-CHS (**B**) or the contralateral side at day 3post-CHS (**D**) have forward flow, but that at day 3 (**C**) on the side of inflammation the lymph flow is perturbed and shows backflow.

Pre-treatment, lymph propulsion occurred in the conducting vessels between the inguinal and axillary lymph nodes at a frequency of 5.3 ± 2.7 pulses/minutes, consistent with previously reported values (See Video S1 and Figure 5A) [12]. The temporal motion can also be visualized using the spatio-temporal fluorescence intensity maps that delineate propagating ICG-laden lymph. In Figure 5A, the region of higher intensity from ICG-laden lymph moves proximally from ROI-1 to ROI-25 in 1.5 sec (arrows on the spatio-temporal plot). At a fixed ROI along the conducting lymphatic vessel (dotted arrow, Figure 5A-D), the fluorescence intensity first increases with the filling of the lymphangion and then decreases, indicating lymphangion contraction and emptying of ICG-laden lymph that is repeated in a peristaltic manner.

At day 3 after onset of CHS, we observed similar rates of contractile lymphatic propulsion of 5.6 ± 2.1 pulse/minutes, but with a consistent “reflux” of ICG-laden lymph (Figure 5B and see Video S2). A typical spatio-temporal fluorescence intensity map at day 3 demonstrates a “reflux” phenomenon with both proximal and distal ICG-laden lymph flow (black arrows, Figure 5B, Figure S2) as well as a broadening of the intensity crest that may indicate incomplete emptying and filling of lymphatic segment. We measured that there was a significant, 2-fold increase in the time it took for the vessel to be filled with dye (the intensity crest) as compared to that in the non-treated animals to the animals at day 3 post-CHS. We also observed that even if the velocity of the lymph (i.e., the time the ICG-laden lymph packet took to move the distance from the inguinal lymph node to the axially LN) was the same. This observation was made in every animal we imaged. These data indicate that the effectiveness of the flow is affected by CHS. At day 7 post-CHS treatment, the lymphatic insufficiency was resolved but still exhibited “reflux” (Video S3 and Figure 5C). The reflux phenomenon was not observed in the contra-lateral, untreated sides of the animals (Video S4 and Figure 5D).

Thus, while the rates of lymphatic contractility were not affected during CHS treatment, lymphatic “reflux” results in less efficient transport of lymph in the inflamed skin lymphatic vessels, resulting in lymphatic insufficiency, with subsequent recovery.

### Identification of pro-angiogenic.- lymphangiogenic factors in CHS

**Figure 6:**
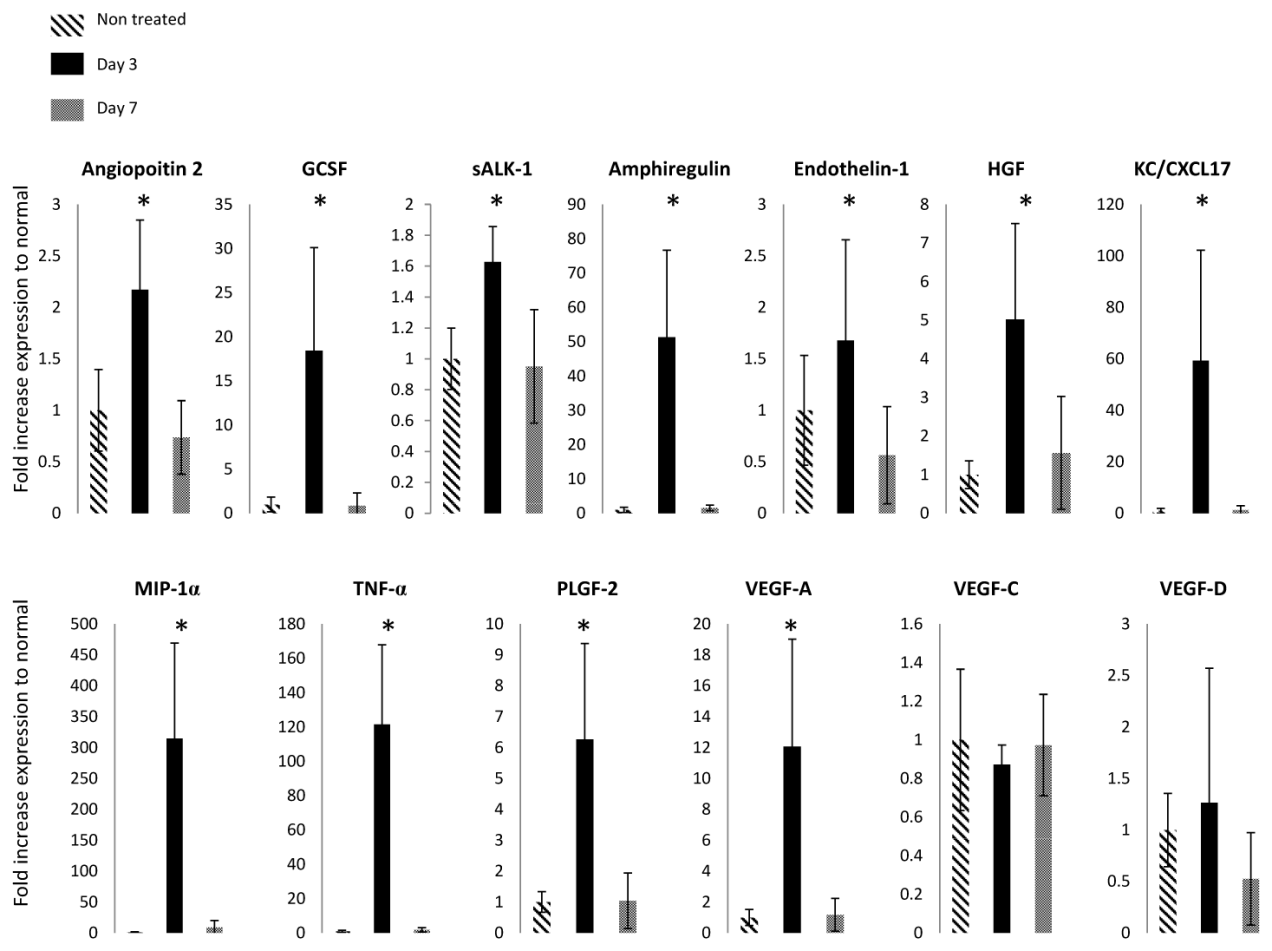
Identification of pro-angiogenic.- lymphangiogenic factors significantly changes in CHS at day 3 post-CHS compared to non-treated and day 7. Multiplex assay was performed on skin homogenate on non-treated, day 3, and day 7 mice post-CHS challenge using the manufacturer’s protocol. We report the mean ratio ±std of the concentration normalized to non-treated values for the analytes that were significantly different. * p<0.05 (FDR<0.1) day3 versus non-treated. # p<0.05 (FDR<0.1) day 3 versus day 7.

We sought to identify which pro-angiogenic and pro-lymphangiogenic factors were present in mice dermal tissues undergoing active LEC proliferation and in which lymphatic function was disrupted. To address the local environment of the lymphatic vasculature, we interrogated the concentration of protein in the dermis and excluded the epidermis which lacks lymphatic vessels. Many growth factors and cytokines are produced by cells in the epidermis (such as keratinocytes), but these factors need to infiltrate the dermis to act directly on LECs. Thus, by assessing the dermis, we measured the growth factors within the local environment of the lymphatic vessels. We performed a milliplex assay for 27 analytes on total protein isolated from mice dermis in non-treated animals, animals at day 3 post-CHS, and day 7 post CHS treatment. We compared the ratios to normal levels between the groups and found 11 factors out of 27 were significantly increased at day 3 compared to both non-treated and day 7 animals (Table S1). The factors, Angiopoietin-2, Granulocyte colony-stimulating factor (G-CSF), sALK-1, Amphiregulin, endothelin, -1, hepatocyte growth factor (HGF), Placenta growth factor- 2 (PLGF-2), macrophage inflammatory protein-1 α (MIP-1α), VEGF-A, TNF-α and KC/CXCL17 were significantly increased (p<0.05 and FDR<0.1) compared to both levels pre-treatment and at day 7 post-CHS (Figure 6). With a sample size powered for 80% and change in ratio of 0.6, it is interesting to note that the concentration of VEGF-C and VEGF-D did not significantly change during the course of CHS reaction with increase/decrease of LEC population and decrease/increase of lymphatic function. This data indicates that other growth factors, which are either increase or decrease in expression in tissues, may work in concert with VEGFC and VEGFD to induce LECs proliferation and changes in lymphatic function in acute CHS.

## Discussion

The phenomenon of lymphatic remodeling or change in lymphatic function caused by acute inflammation is not well understood. In this study, we determined that LECs proliferation occurs in early stages of inflammation and is accompanied by infiltration of CD45+ cells and the loss of lymphatic function.

We developed a method to identify proliferative LECs in vivo to allow us to determine that the LECs proliferate starting at the onset of the CHS, but do not proliferate after the peak of CHS at day 3. The percentage of non-leukocyte cells decreased significantly at day 3 and 7 post-CHS compared to non-treated animals. This decrease in the relative percentage of non-leukocyte cell population is most probably due to the infiltration of CD45+ leukocytes, which is a hallmark of CHS. The relative percentage of BECs within the leukocyte population decreased from non-treated skin to day 3 post-CHS, but may be an artifact in relative numbers due to the increase in infiltrating leukocytes. Others have shown that angiogenesis does not occurs in the ear during CHS since no changes in vessel density were observed, but that the vessels increase in size and become leaky in response to CHS [16]. Here we identified that the BECs population decreases relative to the non-leukocyte population. We hypothesize that this relative decrease in BECs percentage is not due to a decrease in the actual number of cells, but rather due to a reduction of the overall contribution of BECs within the non-leukocyte population that arises because of the proliferation of other non-leukocytes. Using a similar logic, an increase in the relative percentage of LECs within in the non-leukocyte population indicates that the LECs population must change at a higher rate than the average non-leukocyte population and therefore, may be proliferating. Consistent with other reports, our histological results show that the lymphatic density does not increase during CHS [6]. We observed an increase in the area of the lymphatic vessels at day 3 of CHS, which corresponded with the time at which we found that lymphatic vessels become leaky using NIRF imaging. These data are indicative of vessel modification. We also found an increased number of podoplanin+ cells by histology at day 3 post-CHS. The combination of the increase in relative percentage of LECs, the histological observation of the increase number of single podoplanin cells, and the leakiness of the lymphatic vasculature led us to hypothesize that the lymphatic vascular modification includes LEC proliferation. To confirm this hypothesis, we used EdU intercalation as an indication of the level of LEC proliferation. We found that the rate of EdU intercalation increased to 20.45% from 4.25% in non-treated skin, which is indicative of LECs proliferation.

We identified pro-lymphangiogenic factors that are expressed differentially in early CHS, in addition to have tissue expression which parallels the increase and decrease of LEC proliferation and lymphatic function. Our data also suggests that the proliferation of LECs does not lead to the formation of de novo vessels, but rather dilated vessels that could be the cause of the inefficiency of lymphatic contractile pumping. In addition, proliferative LECs may integrate into existing vessels to repair damaged vessels in the inflamed skin. Lymphatic vessels repair possibly requires a dynamic balance between proliferation and vessel regression, similar to that well known and studies in blood vessel angiogenesis [25–27].

We identified that the superficial collecting lymphatic vessels in the inflamed skin regions do not show change in architecture during inflammation, but instead found extravasation of ICG-laden lymph that may be indicative of lymphatic vessel leakiness associated incorporation of with proliferative LECs. Mendez et al. also found that, in acute CHS in the rat foreleg, lymphatic drainage is reduced as shown by interstitial spreading of dye [24].

The peripheral lymphatic system drains excess interstitial fluid back to the blood circulation, but also directs the transport of immune cells. Inflammation impacts lymph drainage and the volume of lymph propelled by lymphatic contraction [23,24]. Herein, we demonstrated that the contractility of the lymphatic vessel efferent to the inflamed area is not affected by the inflammation, but that the effectiveness of the lymphatic pumping is reduced as shown by the backflow observed at day 3 post-CHS and the decrease in ejected dye after contraction. Nitric oxide (NO) is known to affect the lymphatic function through changes in local concentration. Liao et al. proposed that iNOS produced by CD11b+ recruited to the inflamed area promote relaxation of the lymphatic vessels which render the contractions incapable of effectively propelling lymph [23]. Studies in isolated lymphatic vessels have shown that large changes in NO level inhibit contractility and that small change in local concentration influences contraction strength [28,29]. These local changes in NO productions are regulated by the shear forces in the vessel to induce the subsequent contractions [30]. In addition, Scallan et al. found that endothelial NO synthase deficient mice have a decrease in contraction amplitude. In our studies, we observed changes in the contraction amplitude or strength localized to the site of inflammation [28]. This effect was not systemic as indicated by the normal transport of the lymph on the counter lateral, untreated sides of treated animals. Our results support the conclusions of Liao et al. by showing that (i) lymph does not exit lymphangions as efficiently at day 3 post-CHS as compared to pre-treatment and that (ii) this effect is seen only in the inflamed side indicating a local, rather than systematic effect. From our data and the work of others, we can hypothesize that in CHS regulation of local levels of NO, vessel dilation, and shear force within the vessel could regulate the lymphatic flow.

Most studies of lymphangiogenesis have focused on the role of the VEGF family members in this process. Expression of VEGF-A in the skin was found to cause chronic inflammation after induction of CHS which in turned promoted lymphangiogenesis in the inflamed tissue [5]. Both VEGF-C and VEGF-D expression in the skin have been reported to increase the lymphatic vessel size and number [4]. In contrast, Goldman et al. found that VEGF-C did not promote lymphangiogenesis in the skin but had only transient effect on the vasculature [31]. To investigate those factors which are potentially are responsible for promoting LECs proliferation during CHS, we compared the tissue expression levels of proteins at day 3 post-CHS challenge with both non-treated animals and animals at day 7 post CHS treatment. We only included the dermis in our tissue lysate to determine growth factors that locally affect the lymphatic vessels in concordance with LEC proliferation. As observed by Halin et al., we also found an increase in VEGF-A expression in inflamed skin at day 3 as compared to the non-treated animals (Figure 6) [5]. To our surprise we did not observe a significant change in VEGF-C or VEGF-D expression at day 3 compared to non-treated and day 7 post-CHS. In a different model of UVB irradiation, VEGF-C level in the skin was observed to be reduced while the lymphatic vessels enlarged [32]. In our studies, Angiopoietin-2, G-CSF, sALK-1, Amphiregulin, endothelin-1, HGF, PLGF-2, MIP-1α, VEGF-A, TNFα, and KC/CXCL17 were upregulated at day 3 compared to both day 7 post-CHS and in untreated animals. Of the eleven proteins whose profiles followed changes in the LEC proliferation and lymphatic dysfunction, HGF, FGF-2, PLGF-2, Angiopoietin-2, and G-CSF have previously been associated with lymphatic vessel hyperplasia and inducing LECs proliferation [17,33–35]. Interestingly, KC/CXCL17 is a chemokine with anti-inflammatory effects that attenuates the expression of pro-inflammatory genes such as TNF-α and IL-6, as well as iNOS [36]. These factors may act directly or indirectly on the LECs to promote proliferation, vessel remodeling, and influence lymphatic flow potentially in a VEGF-C and -D independent manner.

Our data suggest that lymphatic vessels are remodeled and LECs proliferate in CHS. In addition, CHS induces a local reduction in lymphatic transport through the expression of growth factors and chemokines, which promote vessel dilation and leakiness and/or through the changes in local shear forces. Our study indicates that there is a need to investigate further the importance of other factors such as KC/CXCL17 on lymphatic function and proliferation.

## Supporting Information

Figure S1 **Use of viability Dye Calcein Am is required to identify LECs from skin tissue.** Others have used flow cytometry and lymphatic markers to identify LECs in the tissue, but did not use viability dye to discriminate non cellular events. We found that less than 50% of the events that were the CD45-/CD31+/podoplanin+ did not incorporate Calcein Am. This finding indicates that regular discrimination on Forward and Side scatter are not sufficient to eliminate debris and that the use of viability dyes such as Calcein AM is required to correctly quantify and identify LECs in the skin tissue. We show a sample plot of the events that are podoplanin+/CD31+ which we gated on SSC and FSC and for lack of CD45 staining (left plot). We then selected the double positive events and looked for the percentage of cells that also stained for Calcein AM. We observed that only 35.6% of the cells in the podoplanin+/CD31+ gate are actually live cells.
(PDF)

Figure S2 **Spatiotemporal plots from multiple animals shows lymphatic backflow in CHS.** Plots showing fluorescence intensity along a lymphatic vessel from the inguinal LN to the Axillary LN indicated loss of effective flow at day 3 post-CHS. The plots are from two animals pre-treatment and at day 3 post-CHS. The arrows indicate the direction of the intensity peaks. There is a change in the direction of the intensity peak from always having a forward motion (inguinal LN to the axillary LN) in the pre-treated animals to both forward and backward flow (axillary LN to inguinal LN) in the treated animals. These data are indicative of a change in the effectiveness of the lymphatic flow.
(PDF)

Table S1 **Identification of pro-angiogenic.** **- lymphangiogenic factors in CHS**.
Table shows the mean ratio ±std of the concentration normalized to non-treated values (N>8) of all the analytes assayed and for which the data was reproducible in multiple assay. We also show the ANOVA results, the FDR for the ANOVA, and the corrected p-value for each comparison made.
(PDF)

Video S1 **Video-rate fluorescent imaging of lymphatic flow exposure time of 200 ms.** We recorded fluorescent signal after IGG injection as described in the method section. The video shows 200 frames at an exposure time of 200 ms. The video shows ICG dye packets moving within the lymphatic vessels. Fluorescent signals were converted to pseudo-colored images (Green) using MatLab. Elapsed time from the start of recording is shown in the movie and we show 15 frames per second.
(AVI)

Video S2 **Video-rate fluorescent imaging of lymphatic flow exposure time of 200 ms.** We recorded fluorescent signal after IGG injection as described in the method section. The video shows 200 frames at an exposure time of 200 ms. The video shows ICG dye packets moving within the lymphatic vessels. Fluorescent signals were converted to pseudo-colored images (Green) using MatLab. Elapsed time from the start of recording is shown in the movie and we show 15 frames per second.
(AVI)

Video S3 **Video-rate fluorescent imaging of lymphatic flow exposure time of 200 ms.** We recorded fluorescent signal after IGG injection as described in the method section. The video shows 200 frames at an exposure time of 200 ms. The video shows ICG dye packets moving within the lymphatic vessels. Fluorescent signals were converted to pseudo-colored images (Green) using MatLab. Elapsed time from the start of recording is shown in the movie and we show 15 frames per second.
(AVI)

Video S4 **Video-rate fluorescent imaging of lymphatic flow exposure time of 200 ms.** We recorded fluorescent signal after IGG injection as described in the method section. The video shows 200 frames at an exposure time of 200 ms. The video shows ICG dye packets moving within the lymphatic vessels. Fluorescent signals were converted to pseudo-colored images (Green) using MatLab. Elapsed time from the start of recording is shown in the movie and we show 15 frames per second.
(AVI)
