# Supplementary material for: Lymphatic Vascular Response to Acute Inflammation
Source: PLoS One. 2013 Sep 27;8(9):e76078. doi: 10.1371/journal.pone.0076078 (PMC3785427; doi:10.1371/journal.pone.0076078)
Supplement: Figure S1 — Use of viability Dye Calcein Am is required to identify LECs from skin tissue. Others have used flow cytometry and lymphatic markers to identify LECs in the tissue, but did not use viability dye to discriminate non cellular events. We found that less than 50% of the events that were the CD45-/CD31+/podoplanin+ did not incorporate Calcein Am. This finding indicates that regular discrimination on Forward and Side scatter are not sufficient to eliminate debris and that the use of viability dyes such as Calcein AM is required to correctly quantify and identify LECs in the skin tissue. We show a sample plot of the events that are podoplanin+/CD31+ which we gated on SSC and FSC and for lack of CD45 staining (left plot). We then selected the double positive events and looked for the percentage of cells that also stained for Calcein AM. We observed that only 35.6% of the cells in the podoplanin+/CD31+ gate are actually live cells. (PDF) [file pone.0076078.s001.pdf]

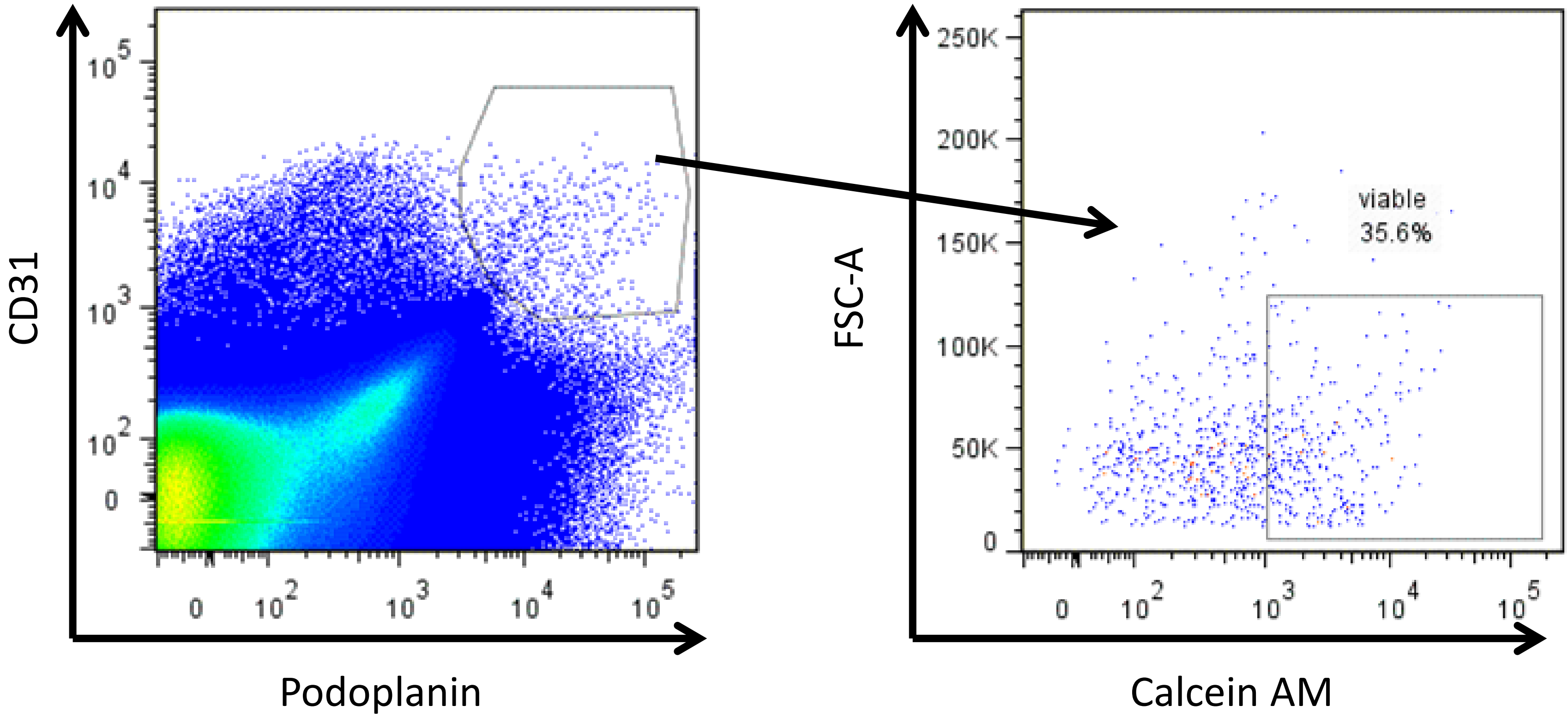

Figure S1

Use of viability Dye Calcein Am is required to identify LECs from skin tissue.
Others have used flow cytometry and lymphatic markers to identify LECs in the tissue, but did not use viability dye to discriminate non cellular events. We found that less than 50% of the events that were the CD45-/CD31+/podoplanin+ did not incorporate Calcein Am. This finding indicates that regular discrimination on Forward and Side scatter are not sufficient to eliminate debris and that the use of viability dyes such as Calcein AM is required to correctly quantify and identify LECs in the skin tissue. We show a sample plot of the events that are podoplanin+/CD31+ which we gated on SSC and FSC and for lack of CD45 staining (left plot).
