# Supplementary material for: Lymphatic Vascular Response to Acute Inflammation
Source: PLoS One. 2013 Sep 27;8(9):e76078. doi: 10.1371/journal.pone.0076078 (PMC3785427; doi:10.1371/journal.pone.0076078)
Supplement: Figure S2 — Spatiotemporal plots from multiple animals shows lymphatic backflow in CHS. Plots showing fluorescence intensity along a lymphatic vessel from the inguinal LN to the Axillary LN indicated loss of effective flow at day 3 post-CHS. The plots are from two animals pre-treatment and at day 3 post-CHS. The arrows indicate the direction of the intensity peaks. There is a change in the direction of the intensity peak from always having a forward motion (inguinal LN to the axillary LN) in the pre-treated animals to both forward and backward flow (axillary LN to inguinal LN) in the treated animals. These data are indicative of a change in the effectiveness of the lymphatic flow. (PDF) [file pone.0076078.s002.pdf]

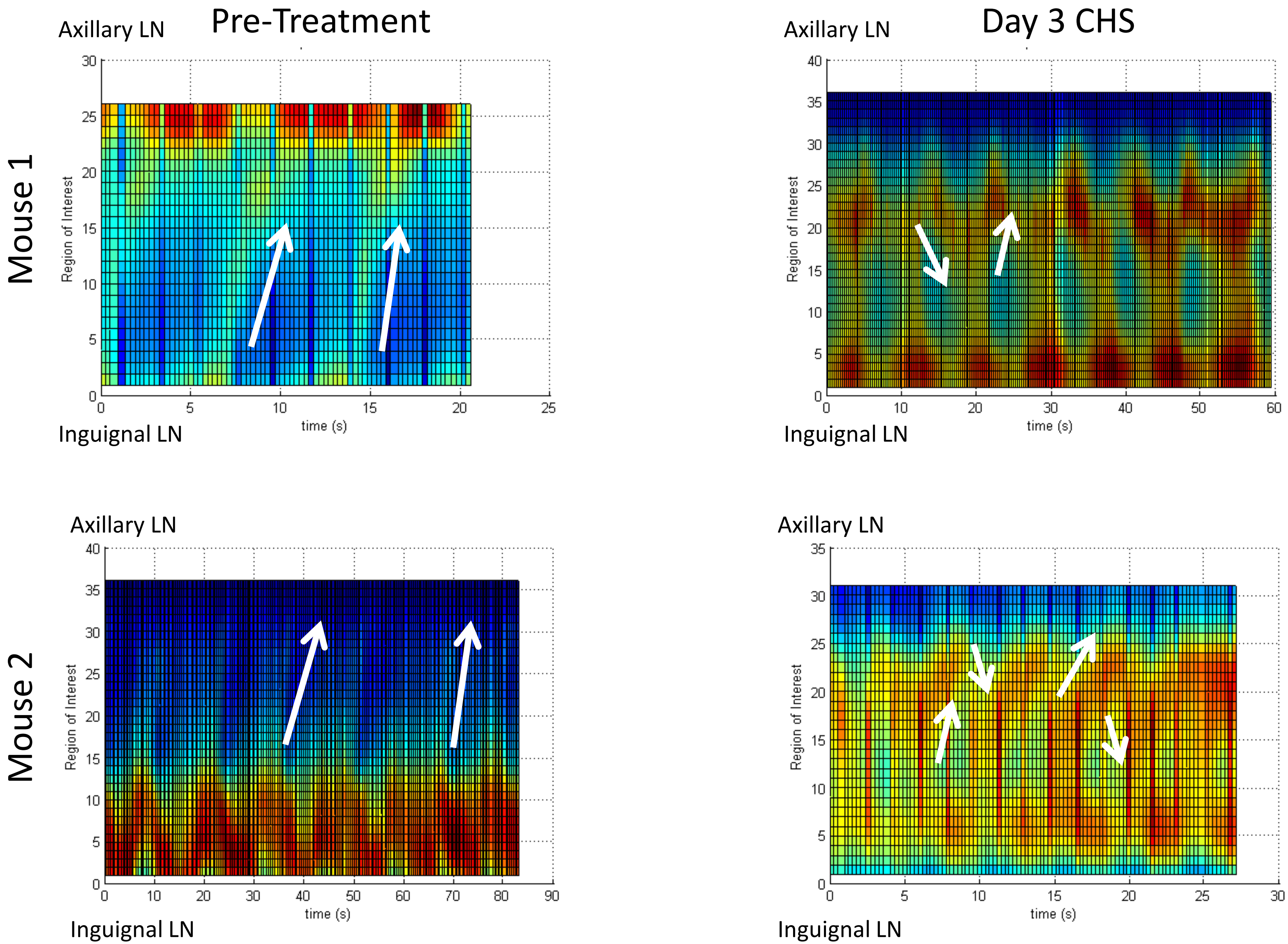

Figure S2

Spatio temporal plots from multiple animals shows lymphatic backflow in CHS Plots showing fluorescence intensity along a lymphatic vessel from the inguinal LN to the Axillary LN indicated loss of effective flow at day 3 post-CHS. The plots are from two animals pre-treatment and at day 3 post-CHS. The arrows indicate the direction of the intensity peaks. There is a change in the direction of the intensity peak from always having a forward motion (inguinal LN to the axillary LN) in the pre-treated animals to both forward and backward flow (axillary LN to inguinal LN) in the treated animals. These data are indicative of a change in the effectiveness of the lymphatic flow.
