# Supplementary material for: Lymphatic Vascular Response to Acute Inflammation
Source: PLoS One. 2013 Sep 27;8(9):e76078. doi: 10.1371/journal.pone.0076078 (PMC3785427; doi:10.1371/journal.pone.0076078)
Supplement: Table S1 — Identification of pro-angiogenic. - lymphangiogenic factors in CHS. Table shows the mean ratio ±std of the concentration normalized to non-treated values (N>8) of all the analytes assayed and for which the data was reproducible in multiple assay. We also show the ANOVA results, the FDR for the ANOVA, and the corrected p-value for each comparison made. (PDF) [file pone.0076078.s003.pdf]

Analyte	normal		Day 3		Day 7		ANOVA		T test value (corrected)		
	ratio to non-treated	std	ratio to non-treated	std	ratio to non-treated	std	P value	FDR	pvalue non-treated vs Day 3	pvalue non-treated vs Day 7	pvalue Day 3 vs Day 7
Angiopoietin-2	1	0.396402	2.171569	0.675327	0.736753	0.356947	7.92E-05	3.06E-04	6.80E-04	3.01E-01	8.50E-05
G-CSF	1	0.824286	18.41181	11.67401	0.856426	1.453234	7.52E-05	3.06E-04	1.80E-04	9.65E-01	1.80E-04
sFASL	1	0.33119	1.277631	0.109582	0.89478	0.494639	3.24E-01	4.37E-01	5.80E-01	6.80E-01	4.70E-01
sALK-1	1	0.199019	1.6274	0.22789	0.950631	0.367444	6.10E-04	1.65E-03	1.74E-03	7.40E-01	9.80E-04
Amphiregulin	1	0.788953	51.31338	25.30838	1.57274	0.822286	1.84E-06	1.65E-05	5.70E-06	9.30E-01	5.70E-06
Leptin	1	0.250236	0.791878	0.604361	0.799216	1.039397	8.38E-01	8.70E-01	1.00E+00	1.00E+00	1.00E+00
Il-1β	1	1.033523	11.84544	19.41941	0.984881	1.76484	1.22E-01	2.53E-01	2.00E-01	1.00E+00	2.00E-01
Betacellulin	1	1.133066	1.721703	0.499555	0.999828	0.933847	4.68E-01	5.49E-01	8.50E-01	1.00E+00	8.50E-01
EGF	1	0.364891	1.044657	0.3819	0.71322	0.527662	3.16E-01	4.37E-01	8.60E-01	5.40E-01	5.40E-01
IL-6	1	0.727628	0.856436	0.312478	0.478503	0.454364	1.75E-01	3.15E-01	6.30E-01	2.20E-01	4.10E-01
Endoglin	1	0.390743	1.35741	0.77241	0.877525	0.812619	4.37E-01	5.36E-01	7.30E-01	7.30E-01	6.40E-01
Endothelin-1	1	0.533426	1.678495	0.97778	0.564466	0.471047	2.17E-02	5.34E-02	1.69E-01	2.24E-01	1.90E-02
FGF-2	1	0.574912	1.538929	0.668173	0.907426	0.723659	2.05E-01	3.47E-01	3.20E-01	7.90E-01	2.80E-01
Follistatin	1	0.96564	0.718267	0.270364	0.406391	0.461321	2.30E-01	3.66E-01	7.60E-01	2.70E-01	7.60E-01
HGF	1	0.359635	5.021043	2.482686	1.565813	1.457815	5.52E-04	1.65E-03	8.30E-04	5.05E-01	1.75E-03
CD31/PECAM	1	0.512695	1.503677	0.884369	0.891083	1.008078	3.88E-01	4.99E-01	5.90E-01	8.00E-01	5.80E-01
IL-17	1	0.583606	2.077666	1.237901	1.483312	2.297605	4.98E-01	5.61E-01	7.30E-01	1.00E+00	1.00E+00
PLGF-2	1	0.335326	6.267112	3.102365	1.044006	0.899691	3.15E-05	2.13E-04	8.40E-05	9.60E-01	8.40E-05
KC/CXCL17	1	0.911678	59.2782	42.92975	1.248528	1.72756	2.06E-04	6.95E-04	4.90E-04	9.83E-01	4.90E-04
MCP-1	1	1.146953	39.97789	55.14014	1.125141	0.912898	4.09E-02	9.20E-02	7.10E-02	9.93E-01	7.10E-02
Prolactin	1	0.309011	0.846046	0.701682	0.880403	0.711766	8.87E-01	8.87E-01	1.00E+00	1.00E+00	1.00E+00
MIP-1α	1	0.759153	314.7234	154.5348	9.117551	10.90567	1.57E-06	1.65E-05	5.40E-06	8.50E-01	5.40E-06
SDF-1	1	0.771958	0.777214	0.762641	0.443893	0.572841	3.22E-01	4.37E-01	7.80E-01	4.20E-01	7.80E-01
VEGF-C	1	0.366127	0.871763	0.101409	0.973124	0.262289	6.81E-01	7.36E-01	1.00E+00	1.00E+00	1.00E+00
VEGF-D	1	0.356655	1.264167	1.305295	0.525236	0.448378	1.42E-01	2.75E-01	5.50E-01	3.40E-01	1.80E-01
VEGF-A	1	0.521792	12.07162	6.943008	1.172247	1.061134	4.22E-05	2.28E-04	1.20E-04	9.30E-01	1.20E-04
TNF-α	1	0.543073	121.4757	46.38383	1.781811	1.234545	3.50E-08	9.45E-07	1.20E-07	9.50E-01	1.20E-07

Table S1

Identification of pro-angiogenic.- lymphangiogenic factors in CHS. Table shows the mean ratio ±std of the concentration normalized to non-treated values (N>8) of all the analytes assayed and for which the data was reproducible in multiple assay. We also show the ANOVA results, the FDR for the ANOVA, and the corrected p-value for each comparison made.
